# Experience of Multisensory Environments in Public Space among People with Visual Impairment

**DOI:** 10.3390/ijerph120808644

**Published:** 2015-07-23

**Authors:** Gavin R. Jenkins, Hon K. Yuen, Laura K. Vogtle

**Affiliations:** Department of Occupational Therapy, School of Health Professions, University of Alabama at Birmingham, Birmingham, AL 35294, USA; E-Mails: jenkinsg@uab.edu (G.R.J.); lvogtle@uab.edu (L.K.V.)

**Keywords:** built environment, public spaces, sensory cues, architectural accessibility, qualitative research

## Abstract

This qualitative study explored the role of sensory characteristics embedded in the built environment and whether they support or hinder people with visual impairment in their use of public spaces. An online survey link was e-mailed to the presidents and committee members of each state’s chapters and associations of the National Federation of the Blind in the United States, resulting in 451 direct invitations to participate. Written responses of the survey questions from 48 respondents with visual impairment were analyzed. Three main themes: Barriers, Supporters, and Context-Dependence emerged from the respondents’ experience of multisensory characteristics within the built environment. The four subthemes subsumed in Barriers were: (1) Population specific design, (2) Extreme sensory backgrounds, (3) Uneven ground surfaces and objects, and (4) Inconsistent lighting. For Supporters, respondents provided specific examples of various sensory characteristics in built environments, including audible cues and echoes, smells, tactile quality of the ground surface, and temperature. Context-Dependence referred to the effects of sensory characteristics embedded in public spaces depending on one’s vision condition, the proximity to the sensory cues and the purpose of the activities one was performing at that moment. Findings provide occupational therapy practitioners an in-depth understanding of the transactional relationship between embedded sensory characteristics in the built environment, occupations, and people with visual impairment in order to make appropriate modifications or removal of barriers that affect occupational performance and engagement. Suggestions for occupational therapists as well as architects, designers, planners, policy makers/legislators related to functional sensory cues in the design of built environments were provided to increase accessibility in the use of public spaces by people with visual impairment.

## 1. Introduction

Study of the dynamic relationship between person, environment and occupation is the core of occupational therapy [1], with occupational performance and engagement and health/well-being the desired outcomes [2]. Environments are not simply places where people want to “access”, instead, they are where many of the occupations that fill people’s lives occur; they are the lexicon of culturally appropriate pursuits and pastimes that provide people with identity and fulfillment [3]. Environments support associations between people and occupations, and contribute to a sense of familiarity and belonging to a community [4]. To understand what people do, there is a need to consider that everyday occupations necessarily occur in particular environments and these environments are related to their occupational engagement [5].

The concept of the environment as a significant factor in promoting people’s ability to engage in activities was the foundation for occupational therapy [6]. Occupation-based theories explain the role of the environment on people’s performance. This is seen in the Canadian Model of Occupational Performance and Engagement, where it is stated that the individual interacts through the medium of occupation with the environment [7]. The Person-Environment-Occupation model asserts that the three elements of person, occupation and environment are in a constant relationship with one another, being characterized as dependent and mutually influential [8]. Accordingly optimal occupational performance may be achieved by changing any of the three components of the model. The environment was also clearly articulated in the Person-Environment-Occupational-Performance, where Christiansen and Baum (1991) proposed that the person and the environment are bridged by occupation and performance [9]. Perhaps most noticeably, within the Ecology of Human Performance the person is portrayed as entirely embedded in the context or environment, depicting an inseparable relationship between the two [10].

Schkade and Schultz (1992) discussed that an environment demands mastery from a person, while the individual desires to behave masterfully [11,12]. The interaction between these two influences results in a dynamic tension between the individual and environment where at times the individual will effect an interaction that is “adaptive and masterful”; while, at other times, they do not. In a similar vein, within the Model of Human Occupation, Kielhofner [13] discussed that in every environment people encounter expectations that demand particular behaviors and discourage others. Each environment offers a number of opportunities and resources, demands and constraints. Aligned with this, other theorists have advocated that a person’s behavior or participation is a response to environmental demands, press or expectations [10,11,12,14] suggesting that cues or conditions in the environment will shape people’s responses. All these occupation-based theories point to the inhibiting or facilitating relationship between individuals’ distinctive performance capabilities, the unique environment in which they function, and the actions and tasks they pursue.

Within the interaction between the person and the environment, Pallasmaa [15] stated that every experience of the environment is multisensory. However, this interaction becomes less clear when a person is not able to draw on the full array of sensory systems available. For persons with loss of vision, constructing an accurate representation of the world is challenging [16]. People with visual impairment need to decode and choreograph an array of sensory interactions in public spaces so they are able to produce an organized and meaningful understanding and awareness of the space around them. This kind of understanding may allow them to effectively navigate through, and more importantly, participate in the activities and services associated with these spaces [16,17,18]. For people with visual impairment, “inaccessibility” is characterized not necessarily by their physical inabilities or the physical form (barriers) of a space, but by information (such as signs), and spatial knowledge that are difficult to access with limited or no vision [19]. For people with visual impairment, this participatory relationship to the “visually biased” public space has been postulated as one of the most challenging interactions between humans and spaces [17].

In addition to tactile and audible sources of information, people with visual impairment perceive olfactory and awareness of motion as sensory cues within an environment that facilitate orientation and safe and accurate mobility in public spaces [20,21,22,23]. Studies on sensory interactions in public spaces among individuals with visual impairment have focused mainly on how these individuals adapt to different urban or built environments [21,22,23,24]. The built environment refers to “spaces such as buildings and streets that are deliberately constructed as well as outdoor spaces that are altered in some way by human activity” ([25], (p. 1591)). These studies explored the different sensory cues that people with visual impairment used most often for wayfinding in urban or built environments [21,22,23,24].

However, findings of a recent scoping review of 22 articles, which included 15 qualitative and seven quantitative research designs published between 1997 and 2010 on factors associated with activity participation and engagement among adults with low vision, indicated few studies have explored the influence of environmental factors on activity participation and engagement in this population [26]. This is despite available evidence suggesting that the level of participation among people with visual impairment in activities varies based on characteristics of the environment [17].

Since the environment in which individuals with visual impairment interact plays a central role as either a barrier or facilitator of activity participation and engagement [18], exploring the experience of the sensory characteristics in the public spaces among these individuals may provide crucial information. The purpose of this study was to explore the experience of sensory characteristics in public spaces among people with visual impairment residing in the United States.

## 2. Methods

### 2.1. Research Design

This descriptive study involved a cross-sectional survey research design. The study was approved by the Institutional Review Board of the University of Alabama at Birmingham. Implied consent was provided by participants upon submission of the completed survey.

### 2.2. Participants

Participants eligible for study inclusion were presidents and committee members of each state’s chapters and associations of the National Federation of the Blind (NFB, https://nfb.org/state-and-local-organizations). The NFB is the largest organization of blind and low-vision people in the United States, and has affiliates in all fifty states, the District of Columbia, and in Puerto Rico.

### 2.3. Instrument

The survey contained nine closed-ended and two open-ended questions. The closed-ended questions asked the respondents with visual impairment to rank the importance of 40 distinctive sensory characteristics in the built environment that might allow them to successfully use public spaces. Quantitative findings of these questions were reported elsewhere [20]. The present study focused on the responses to the two open-ended questions in the survey (1) “Please share any experiences of multisensory environments or particular environments that are either supportive or inhibiting to you” and (2) “Please provide your opinion on creating built environments that capitalize on sensory cues such as sound, textures, smell and others, as a means to support the use of public spaces.” The initial version of the survey was sent to a small group of orientation and mobility specialists and the Professional Development and Research Institute on Blindness, Louisiana Tech University for content and structure reviews; changes were then made to the questions and survey construction to improve accessibility for people with visual impairment.

### 2.4. Procedures

The survey was posted on Survey Monkey™ (Surveymonkey.com, Portland, OR, USA), an online survey website engine, which provided a Uniform Resource Locator (URL) for the survey. The survey instrument URL and a cover letter explaining the purpose of the survey were e-mailed to the presidents and committee members of NFB chapters and associations for each state and the District of Columbia, excluding Puerto Rico. A total of 451 direct invitation e-mails were sent. Participation in responding to the survey was voluntary with no incentive other than contributing to general knowledge.

Through delivery of the specific platform in SurveyMonkey™, and screen reader software that reinterprets graphical user interfaces and communicates the content by refreshable braille or voice synthesis [27], individuals with visual impairment could respond to the survey. After the initial email and a follow-up email, we received 85 responses, with a response rate of 18.8%. Data were collected between the beginning of June and the first week of October 2011. These 85 responses came from 34 states. Of the 85 respondents, 48 responded to either one or both of the two opened-ended questions; 41 provided their opinions on creating built environments that capitalize on sensory cues, 29 provided written responses on their experience of multisensory environments, and 22 provided responses to both questions.

### 2.5. Thematic Content Analysis

Three investigators (Gavin R. Jenkins, Laura K. Vogtle, and Hon K. Yuen) used the following steps to analyze the content of the written responses. Two investigators (Laura K. Vogtle and Hon K. Yuen) were not involved in the original design of the questionnaire. The three investigators independently read all written responses several times to gain an overall impression of the content, and to formulate tentative ideas. Initial codes were manually assigned to phrases and sentences; they were closely associated with the original text in order to maintain the respondents’ meaning. Using an ongoing process of comparing text segments across the written responses, the investigators sought similarities or repeated ideas using the constant comparative method; codes were refined, revised and added as new topics emerged [28]. Codes expressing related concepts were grouped together to create categories, then collapsed into themes and subthemes [29]. To enhance the credibility of the analysis, all three investigators reviewed each other’s data interpretation and categorization, and compared and contrasted the findings. When there were disagreements, we reviewed the respondents’ written responses, then discussed, resolved disagreements, and reached consensus. Several rounds of discussion were used to develop categories, themes and subthemes that described respondents’ experience of sensory characteristics in public spaces. Data triangulation was conducted by comparing the respondents’ ranking on the importance of 40 distinctive sensory characteristics in the built environment that might allow them to successfully use public spaces.

## 3. Results

Based on the analysis of the 70 (*i.e.*, 41 + 29) written responses of the two open-ended questions from 48 respondents who responded to either one or both of the two opened-ended questions, we identified three main themes: Barriers, Supporters, and Context-Dependence. Four subthemes subsumed in the theme of Barriers that respondents with visual impairment encountered in multisensory environments were: (1) Population specific design, (2) Extreme sensory backgrounds, (3) Uneven ground surfaces and objects, and (4) Inconsistent lighting.

### 3.1. Barriers

#### 3.1.1. Population Specific Design

As indicated by the respondents, often times the built environment is constructed from the perspective of sighted persons with the goals of convenience, artistry (visual appeal with little functional purpose), and style without considering that some features (e.g., low hanging signs) can serve as barriers that exclude people with visual impairment from accessing the environment or even posing safety hazards to them. Below are some of the quotes from three respondents which illustrate the barriers for people with visual impairment when the environments include population specific design.

R5: The popular choice nowadays is very open and spacious areas. These offer we blind much less audio and so forth information. In fact in places like hotel lobbies or large halls the vaulted ceiling that are used can confuse the sound information and in fact magnify it to much higher levels.

R25: Too often people plan environments to be visually appealing without even considering some simple options that would still be visually appealing, but would allow for more independence to a person with a vision loss.

R25: In traveling, curb cuts that point to the center of the intersection rather than having 2 separate curb cuts to cross each street are difficult to cross correctly.

R37: As an example of an environment that works well for the sighted, but is not blind friendly, the central station of our metro bus system. … The buses line up in two lines with a central patio-like area in the middle with a roof over it. … They [the sighted] can jump off one bus, cross the plaza area, then hop onto another bus. For the blind, the setup is a nightmare. With ten buses together, engines running under a roof, it’s like being inside a drum with someone pounding on it. As one totally blind, I rely almost solely on sound. I cannot distinguish one bus from another, and the cacophony of noise seems to jumble my mobility skills. Without sighted help, it's just about impossible for me to go from one bus in one line to a bus in another line. Even to talk within this environment with all buses there is extremely difficult.

#### 3.1.2. Extreme Sensory Backgrounds

Extreme sensory backgrounds such as loud persistent noises that mask target signals or desired useful sensory (audible) information (e.g., in street crossing) can affect orientation and mobility for directional travel, as well as pose potential safety risks. Likewise, an environment that has significant echo with reverberating noise can create sound distortion. This makes it difficult to isolate sound cues and determine the direction of the source of the sound in locations where people with visual impairment rely on auditory cues for travel. On the other hand, large open spaces, such as open fields and parking lots that are void of any sensory cues, are also difficult to navigate. Similarly, hybrid vehicles that produce very little noise or the absence of parallel traffic surges at street crossings can confuse people with visual impairment as to when to start crossing the street. Below are quotes from two respondents, which illustrate the barriers for people with visual impairment when the environments have extreme sensory backgrounds.

R1: Hybrid vehicles are very dangerous for the blind person who depends on the parallel traffic surge to know when to start crossing, how to keep aligned in or near the crosswalk, and just that the street is clear of oncoming vehicles.

R40: Also in certain buildings, it is difficult to determine the direction of sound if the noise is reverberating.

#### 3.1.3. Uneven Ground Surfaces and Objects

Uneven ground surfaces such as cracks, bumps, and drop-offs on the sidewalk can create fall risks. Quotes from two respondents illustrated the potential hazards of uneven ground surfaces and objects in the environment imposed on them.

R1: …tree with big roots that may be pushing up the sidewalk and causing dangerous uneven surfaces or drop-offs…

R6: Low lying obstacles, like signs are potential hazards that can cause injuries to people who are blind.

#### 3.1.4. Inconsistent Lighting

Finally, inconsistent lighting, which casts shadows, and low contrast signage, can confuse people with visual impairment. One respondent (R41) commented “Trees or other buildings that cast a shadow at step downs cause great trouble for those who have minimal sight or light perception only.”

Some of these identified barriers such as inconsistent lighting and uneven ground surfaces and objects, as well as certain features in population specific design, were commonly reported in the literature as impediments for travel in people with visual impairment [16,23,30,31,32]. While other barriers such as extreme sensory background, and population specific design were unique as reported by participants in this survey.

### 3.2. Supporters

Specific sensory cues associated with the auditory, olfactory and somatosensory (haptic and temperature) systems are important to people with visual impairment for orientation and mobility to allow them to participate in various activities. However, such sensory cues need to have specific purpose and meaning to them. Respondents provided specific examples of various sensory characteristics in built environments, including audible cues and echoes, smells, tactile quality of the ground surface, and temperature. These examples were represented by the four subthemes: Temperature cues, Haptic cues, Olfactory cues and Auditory cues, that allowed participants successful use of public spaces. For example, temperature, airflow direction and placement of the sun relative to the individual (*i.e.*, Temperature cues), and textured landmarks (*i.e.*, Haptic cues) which guide them to a point of interest and indicating directions of travel were mentioned by respondents as supports to their orientation and mobility. These were illustrated by the following quotes from three respondents.

R4: Cold air in a warm building plus traffic sounds may indicate an open door to the outside.

R27: …steam came up from grates in the streets or sidewalks, this could be important [for travel].

R21: A rough texture to a certain driveway or sidewalk path can also act as landmarks to and from particular places.

Smell associated with places can assist orientation, and verify one’s location or travel path, as it creates a distinct landmark for a particular area (*i.e.*, Olfactory cues). As one respondent (R39) remarked “Bathroom deodorizers are sometimes good cues.” Audible pedestrian signals, echoes from buildings and other objects, and landmark sounds (e.g., fountains or traffic) help travelers with visual impairment orient and navigate public spaces. These were illustrated in the follow two quotes.

R10: Audible pedestrian signal with beeps and chirps give directional crossing cues before crossing intersections.

R28: …echoes from buildings and other objects to help us know where we are and where we are headed.

Utilization of these sensory cues for wayfinding was consistent with those used by people with visual impairment in previous studies [21,22,23]. Often respondents with visual impairment reported that they capitalized on multi-sensory cues (such as traffic sounds, smell, texture of ground surface, and temperature) collectively to determine and validate their direction for orientation and mobility [31].

### 3.3. Context-Dependence

Respondents also argued that the effect of sensory cues in public space depended on the characteristics of the sensory input (type, intensity, pitch, and tone), the proximity to the sensory cues, the residual vision of the person (as one respondent (R1) noted that “…depending on the eye condition of the individuals, bright light helps some people and even dull lighting helps others.”), and the type and nature of the activities (e.g., directional orientation *versus* identification). For example, loud noise, if close to the individual with visual impairment, is problematic because it is likely to mask desired useful sensory (audible) information. However, if the loud noise is far away, it can serve as a directional sound for orientation. In the same vein, quiet sounds (like modern hybrid cars), if close to the individual with visual impairment, may be dangerous, as these sounds cannot be easily heard. Whilst quiet sounds are of little importance if they are far away. These were illustrated by the following two quotes.

R11: Loud traffic sounds can be helpful for determining walking directionality, and at the same time problematic for identifying an approaching bus.

R77: … loud (masking sounds if close are problematic) but if far are important. …quiet sounds (if close and for example a car) are very problematic as they cannot be easily heard—but would be of little importance if far away...

## 4. Discussion

Understanding the dynamic interplay of real world factors that influence use of public spaces by people with visual impairment is essential to facilitate full participation by this population. Shedding light on the role that sensory cues play in the experience for people with visual impairment, will advance understanding of the association between people with vision loss, spaces and levels of participation. People with visual impairment rely on a personal and unique combination of sensory inputs to produce an organized and meaningful understanding and awareness of the spatial experience of public spaces. Congruence between the information processing of people with vision loss and sensory cues in the environment are needed if they are to be successful in using places and spaces. If these sensory signals are effectively comprehended, they will allow the individual to understand and use that space to promote participation and activity [16,18]. If these signals conflict, the result may be uncertainty, fear, distress, discomfort and more often a loss of performance and participation.

Occupational therapy is the pivotal profession in the rehabilitation and habilitation of people with visual impairment and blindness [33,34]. Acknowledging the collaboration of sensory systems of sound, taste-smell, basic-orientating and haptic perception to compensate for loss of vision and define the interaction between the person and the space is critical in occupational therapy’s role as the profession to bridge the gap between “accessibility” initiatives and fully support participation and engagement in the real life experiences of public spaces. Furthermore, to be effective in assisting clients with visual impairment in occupational performance and engagement, occupational therapy practitioners must possess an in-depth understanding of the transactional relationship of embedded sensory characteristics in built environments, occupations, and people with visual impairment to make appropriate modifications or removal of barriers that affect occupational performance and engagement [1,34].

Findings from this qualitative analysis also provide several tangible ideas for architects, designers, planners, as well as policy makers/legislators, to consider when incorporating functional sensory cues in the design process of public space that are user-friendly to this population. The goal is to provide people with visual impairment with accurate orientation, safe mobility travel and wayfinding, and to support their participation in activities and services present in public space.

For outdoor spaces, more attention should be paid to sound and surface texture for safe street crossings, which includes installation of accessible pedestrian signals at traffic light intersections and vehicle detection devices, especially for quiet running hybrid vehicles. In addition, installation of tactile ground surface indicators (such as truncated domes) to assist wayfinding and provide warning of upcoming dangers (e.g., edges of railway platforms), changes in traffic direction (e.g., when crossing roads), and integration of appropriate landmarks cues for inclines, declines, turns, curves, and even suitable placement of street furniture are recommended.

For indoor spaces, information sources about the building should be available in auditory and/or tactile format, such as tactile maps with auditory feedback or labeled with braille, for topographical location. Directional signage should be large with bright lettering as effective orientation cues. Consistent, well-lit, non-florescent lighting, without glare, and high luminance for contrast are important. Reduction of non-directional background noise and echo reverberation generated by ventilation fans and machines can be achieved by having acoustic tiles on the ceiling and other soft items on the walls. Strategic placement of certain sound emitting objects, such as fountains, which provide landmark sounds and directional information about the layout of an interior space, are recommended. Using contrasting textures to define different areas, and unique textures for landmarks are important. Floor surface materials that provide distinct tactile and auditory feedback for cane tapping is critical for accurate traveling. Airflow inside the building and control of air quality and temperature (e.g., smell and sunlight) can assist with orientation and travel direction.

In the end, the key is to generate a balance of salient sensory cues in the built environment. It may not be feasible, for example, to have different floor surfaces or an announcement at every entry of each facility or store to tell people where they are and at every location while they are in a building. However, within the public domain there can be compromises achieved between forcing issues to accommodate certain populations, a strategy that itself may create stigma, discrimination, and alienation, and designing public spaces to be engaging and welcoming to a broad spectrum of possible users.

The underlying philosophy of universal design on the form and function of public space may hold the solution [35,36]. Universal design is “the design of products and environments to be usable by all people, to the greatest extent possible, without the need for adaptation or specialized design” (p. 1) [37]. The conscious act of designing public spaces with strategically or creatively embedded sensory cues in the built environment has the potential to support the fair use of public spaces, and the concept of shared space [30,38,39,40]. If done creatively, this process could support all possible users in their participation and engagement of activities, services, and facilities, without identifying or stigmatizing a particular group of individuals, such as people with visual impairment, in the use of public spaces. When designing the layout of space or planning the configuration of services and facilities of a building, it is important for the architect or designer to consciously design the built environment so that it will not have any significant negative impact or cause inconvenience to individuals who use that space, including people with disabilities. Often, subtle changes to building layout can make a significant positive impact on people with visual impairment but require only minimal adaptation for other users. Such subtle changes may, however, require conscious involvement of a particular group of persons with disability, such as individuals with visual impairment, in the design stages [38]. Therefore, involvement of potential users with disabilities such as visual impairment, as well as rehabilitation professionals, such as occupational therapists, in built environment design is vital.

As one respondent from the state of Florida succinctly summarized “If parks and paths had some audible cues to let the blind person know when they had strayed away from the path BEFORE the inevitable fall at the drop-off point, we could go to the park on our own. If city planners included the use of talk to speech technology like iPhones, we could cross the streets safely and enjoy a greater measure of freedom. When the “silent” cars all make the same sound to warn the blind of their presence, we will be safe on our streets just like you are. When all of the appliances in an office or home have speech functionality along with the lcd (liquid-crystal display) displays in use today, we will be able to hold jobs and operate equipment just like you.”

Using a thorough ergonomic work analysis assessment in work situations, occupational therapists can provide tailored interventions for clients with visual impairment to meet the physical and social demands of the workstation. Interventions may include adjusting and rearranging the work-based environment, providing specialized assistive technology devices that enhance and/or substitute vision to access work-related information or the work environment, and teaching the client to use compensatory strategies for job/task specific duties [41,42].

However, beyond the structural modification and design of the built environment, awareness and public attitudes towards people with disabilities, including those with visual impairment, need to be considered [32,43]. The strategy of embedding sensory cues into the built environment will only reach their true potential when other users of the environment, out of courtesy and respect of the person with a disability, modify their behaviors. Noting a person with a visual impairment and momentarily stopping the lawn mower or leaf blower when the person is trying to listen to the accessible pedestrian signal and cross the street may not truly affect productivity, but for that person with visual impairment, it will support his/her independent participation.

Several strengths of this study are worthwhile for highlighting. First, a unique sampling strategy was used to recruit participants with visual impairment. Capitalizing on the membership database of the state’s chapters and associations of the National Federation of the Blind, we were able to recruit participants with visual impairment from 34 states in the US. Second, an investigator triangulation approach was used in cross-checking and verifying the interpretation of the data [44]. Investigator triangulation involved two authors (LKV and HKY), in addition to the lead author (GRJ), who conducted the data analysis, cross-checked the plausibility of the data interpretation and discussed the meaning of the categories until consensus was achieved [45]. Third, the concept of context-dependence emerged from this study provides a unique perspective of the effects of sensory characteristics embedded in public spaces for health care professionals such as occupational therapists, as well as architects, designers, and planners to consider when designing or modifying built environments.

### Limitations

Even though responses of this survey came from 34 states in the US, of diverse geographic regions, we did not know how representative our data are. The results may be skewed as we only recruited people with visual impairment who have access to technology. Further studies should validate the findings by including people with visual impairment who do not use technology. Finally, we did not collect any social characteristics and information on the severity of visual impairment of the respondents. As a result, we did not know how each category of social characteristics and severity of visual impairment may shape the multisensory experience of the respondents. A purposive sampling of people with visual impairment from orientation and mobility training services and low vision clinics, with specific residual visual condition, diagnostic group, and socio-demographic background can enrich the trustworthiness of the findings.

## 5. Conclusions

Based on the qualitative synthesis of the participants’ responses, it was concluded that experience of multisensory environments from the perspective of people with visual impairment are not just supportive or inhibiting to their activity participation and engagement, but are also context-dependent. Context-dependence may encompass characteristics and proximity of the sensory cues, type and nature of the activities, and the residual vision of the person. Suggestions for architects, designers, planners, and policy makers/legislators to adopt the principles of universal design on form and function when designing indoor and outdoor public spaces were provided so as to increase accessibility in the use of public spaces by all possible users, including people with visual impairment. Findings from this qualitative analysis also provided occupational therapy practitioners an in-depth understanding of the transactional relationship of embedded sensory characteristics in built environment, occupations, and people with visual impairment to make appropriate modifications or removal of barriers that affect occupational performance and engagement.
